# Impact of Backbone Amide Substitution at the Meta- and Para-Positions on the Gas Barrier Properties of Polyimide

**DOI:** 10.3390/ma14092097

**Published:** 2021-04-21

**Authors:** Qian Wen, Ao Tang, Chengliang Chen, Yiwu Liu, Chunguang Xiao, Jinghua Tan, Duxin Li

**Affiliations:** 1 State Key Laboratory of Powder Metallurgy, Central South University, Changsha 410083, China; wenqian0088@126.com (Q.W.); chunguang4715@126.com (C.X.); 2 Key Laboratory of Advanced Packaging Materials and Technology of Hunan Province, National and Local Joint Engineering Center of Advanced Packaging Materials R & D Technology, School of Packaging and Materials Engineering, Hunan University of Technology, Zhuzhou 412007, China; tangao1234@163.com (A.T.); chenchengliangc@163.com (C.C.); tjh@hut.edu.cn (J.T.)

**Keywords:** polymerisation, flexible display technology, dianiline monomer

## Abstract

This study designed and synthesised a meta-amide-substituted dianiline monomer (m-DABA) as a stereoisomer of DABA, a previously investigated para-amide-substituted dianiline monomer. This new monomer was polymerised with pyromellitic dianhydride (PMDA) to prepare a polyimide film (m-DABPI) in a process similar to that employed in a previous study. The relationship between the substitution positions on the monomer and the gas barrier properties of the polyimide film was investigated via molecular simulation, wide-angle X-ray diffraction (WXRD), and positron annihilation lifetime spectroscopy (PALS) to gain deeper insights into the gas barrier mechanism. The results showed that compared with the para-substituted DABPI, the m-DABPI exhibited better gas barrier properties, with a water vapour transmission rate (WVTR) and an oxygen transmission rate (OTR) as low as 2.8 g·m^−2^·d^−1^ and 3.3 cm^3^·m^−2^·d^−1^, respectively. This was because the meta-linked polyimide molecular chains were more tightly packed, leading to a smaller free volume and lower molecular chain mobility. These properties are not conducive to the permeation of small molecules into the film; thus, the gas barrier properties were improved. The findings have significant implications for the structural design of high-barrier materials and could promote the development of flexible display technology.

## 1. Introduction

Polyimide (PI) is a class of polymeric material with excellent comprehensive performance [1,2]. PI demonstrates outstanding thermal and mechanical properties, good dimensional stability, and excellent electrical insulation due to its unique imide ring structure [3]. Moreover, the dianiline/dianhydride monomer of polyimide can be designed and synthesised flexibly and easily [4]. Further, their raw materials are diverse and inexpensive, leading to PI’s excellent structural designability. PI is widely used in many fields, including aerospace, machinery, electronics, and microelectronics [5,6].

The advancement of new technologies within microelectronics has posed increasingly high requirements for the properties of polymeric materials. For example, the polymeric substrate materials used in the packaging of flexible organic light-emitting devices (OLED) must withstand temperatures up to 400 °C and have a permeability several orders of magnitude lower than that of traditional high-barrier materials [7,8,9]. While such strict performance requirements cannot be achieved by traditional high-barrier polymeric materials, the excellent heat resistance of PI makes it a promising substrate candidate [10,11]. However, the application of traditional PI as an OLED substrate is limited by its low barrier properties [12,13]. Fortunately, it is possible to synthesise PI with high barrier properties thanks to its excellent structural designability [14,15]

**Figure 1:**
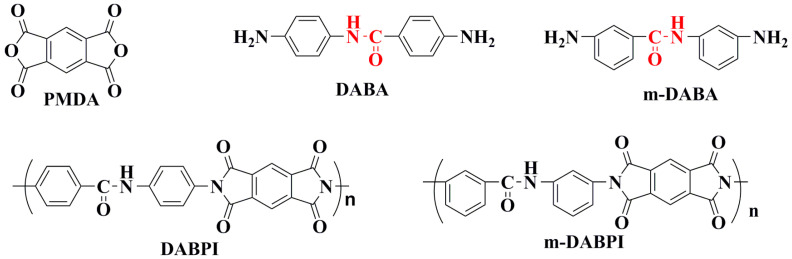
The chemical structure of PMDA, DABA, 3-amino-N-(3-aminophenyl)benzamide (m-DABA),4-methyl-N-(4-(6-methyl-1,3,5,7-tetraoxo-3,5,6,7-tetrahydropyrrolo[3,4-f]isoindol-2(1H)-yl)phenyl)benzamide (DABPI), and 3-methyl-N-(3-(6-methyl-1,3,5,7-tetraoxo-3,5,6,7-tetrahydropyrrolo[3,4-f]isoindol-2(1H)-yl)phenyl)benzamide (m-DABPI).

Relevant studies have shown that chemical structural design is among the most effective ways to improve the intrinsic barrier properties of PI. Existing studies have focused on introducing rigid planar (carbazole, fluorene rings, etc.) or amide structures that can realise interchain forces to prepare high barrier PI with excellent performance [16,17,18]. In our previous work, the high barrier PI was generated by the reaction of a commercial p-diamine monomer (DADA) containing an amide group with commercial pyrophthalic anhydride (PMDA). However, much remains unknown about the impact of subtle changes in monomer structure on the gas permeation properties of such polymers [19,20]. This study synthesised a meta-substituted dianiline monomer (m-DABA; see Figure 1 for its structure) via amidation and reduction based on previous research on a para-amide-substituted dianiline monomer (DABA; see Figure 1 for its structure) [18]. These two monomers are stereoisomers of each other, which enables us to further study the effect of spatial isomerism on the tight accumulation of PI molecular chain and gas permeability.

Next, m-DABA was polymerised with commercial pyromellitic dianhydride (PMDA, see Figure 1 for its structure) to prepare a PI film (m-DABPI, see Figure 1 for its structure) in a process similar to that for the DABA-based PI film. The thermal, mechanical, and barrier properties of the new film were tested. Wide-angle X-ray diffraction, positron annihilation lifetime spectroscopy (PALS), and molecular simulation were conducted to analyse the aggregation structure, free volume, chain mobility, molecular motion trajectory, diffusion coefficient, and solubility coefficient of the two polyimide materials (DABPI vs. m-DABPI), providing a detailed systematic exploration of the impact of substitution position on the barrier properties of PI. In summary, we try to change the spatial isomerization structure of monomers to develop a higher barrier polymer film, and the barrier mechanism is revealed.

## 2. Materials and Methods

### 2.1. Materials

m-nitroaniline, palladium 10% on activated carbon (10% Pd/C), hydrazine monohydrate (N_2_H_4_∙H_2_O), 3-nitrobenzoyl chloride and 4,4′-Diaminobenzanilide (DABA) were purchased from Alfa-Aesar company and used as received. PMDA was purchased from Alfa-Aesar company and dried at 110 °C under vacuum for 6 h before use. Analytical grade absolute ethanol (EtOH), N,N-dimethylformamide (DMF), N-N-dimethylacetamide (DMAc), and pyridine (Py) were purchased from Sinopharm Chemical Reagent Co., Ltd. DMF was purified by distillation under inert nitrogen atmosphere. DMAc and Py were dried by 4A molecular sieve for a week before use. Absolute ethanol was used without further purification.

### 2.2. Instrumentation

All characterisation methods are shown in the Supporting Information.

### 2.3. Synthesis of 3-Nitro-N-(3-Nitrophenyl)Benzamide(m-DABN)

m-nitroaniline (6.906 g, 50 mmol) was placed in a 250 mL three-necked flask into which 100 mL of dry N,N dimethylacetamide (DMA_C_) and 14 mL of pyridine (Py) were sequentially added. The reactor was fully deoxygenated, filled with argon, and stirred at low temperature for 10 min. The resulting mixture was slowly added to m-nitrobenzoyl chloride (10.206 g, 55 mmol), followed by 24 h stirring at room temperature. Next, the reaction mixture was poured into 2 L of water to allow for precipitate formation. After thoroughly washing the precipitate, the greyish-white product was collected and vacuum-dried at 80 °C for 24 h with a yield of 80% (11.49 g). IR (KBr, *v*, cm^−1^): 1527 (−NO_2_ stretching), 1652 (C=O stretching), 1347 (C−N stretching), 3307 (N–H stretching), 1100 ~ 700 (Ar–H stretching).^1^H NMR (400 MHz, DMSO-*d_6_*, *δ*, ppm): δ 10.99 (s, 1H), 8.88 (t, *J* = 27.6 Hz, 1H), 8.81–8.73 (m, 1H), 8.52–8.36 (m, 2H), 8.22 (d, *J* = 7.6 Hz, 1H), 8.11–7.93 (m, 1H), 7.95–7.80 (m, 1H), 7.69 (t, *J* = 8.2 Hz, 1H). ^13^C NMR (100 MHz, DMSO-*d_6_*, *δ*, ppm): 164.24, 148.33, 148.23, 140.34, 135.98, 134.74, 130.77, 130.61, 127.03, 126.80, 122.94, 119.02, 115.06. MS (EI, *m*/*z*): 288(100) ([M^+^ + H], calcd for C_13_H_9_N_3_O_5_, 287.05). Anal. calcd for C_13_H_9_N_3_O_5_: C, 54.36; H, 3.16 and N, 14.63; found: C, 54.31; H, 3.19 and N, 14.61.

### 2.4. Synthesis of 3-Amino-N-(3-Aminophenyl)Benzamide (m-DABA)

m-DABN (2.872 g, 10 mmol) was placed in a three-necked flask into which 200 mL of absolute ethanol was added. The resulting mixture was fully deoxygenated and then filled with argon. After increasing the reactor temperature to 80 °C, a carbon-supported palladium (Pd/C) catalyst (0.02 g) was added, followed by the dropwise addition of 10 mL hydrazine hydrate to allow the reaction to take place under the argon atmosphere at 80 °C for 5 d. Then, the reaction was terminated, and the reactor was allowed to cool to room temperature, followed by the vacuum filtration of the reaction mixture to remove the catalyst. The filtrate was rotary evaporated to a minimum amount and then allowed to crystallise at a low temperature to obtain a crystalline dianiline monomer, which was vacuum dried at 80 °C for 24 h for a yield of 85% (1.932 g). IR (KBr, *v*, cm^−1^): 3419 (N–H stretching), 3336 (–NH_2_ stretching), 1621 (*δ* N–H), 1645 (C=O stretching), 1257 (C–N stretching), 1100 ~ 700 (Ar–H stretching).^1^H NMR (400 MHz, DMSO-*d_6_*, *δ*, ppm): 9.89–9.67 (m, 1H), 7.15–7.10 (m, 2H), 7.08 (d, *J* = 1.5 Hz, 1H), 7.06–7.01 (m, 1H), 6.95 (t, *J* = 7.9 Hz, 1H), 6.84 (dd, *J* = 9.9, 9.2 Hz, 1H), 6.73 (ddd, *J* = 15.7, 8.5, 7.2 Hz, 1H), 6.35–6.23 (m, 1H), 5.17 (d, *J* = 103.1 Hz, 4H). ^13^C NMR (100 MHz, DMSO-*d_6_*, *δ*, ppm): 166.63, 149.30, 149.13, 140.40, 136.81, 129.20, 129.16, 117.04, 115.22, 113.48, 110.04, 108.83, 106.53. MS (EI, *m*/*z*): 228(100) ([M^+^ + H], calcd for C_13_H_13_N_3_O, 227.11). Anal. calcd for C_13_H_13_N_3_O: C, 68.70; H, 5.77 and N, 18.49; found: C, 68.61; H, 5.79 and N, 18.52.

### 2.5. Synthesis of Polyimides (m-DABPI)

In a clean room, m-DABA (0.5454 g, 2.4 mmol) was placed in a round-bottomed flask into which 10 mL of dry N,N’-dimethylformamide (DMF) was added to dissolve it, followed by the addition of PMDA (0.5235 g, 2.4 mmol) with a solid content of 10 wt.%. After filling the reactor with argon, the mixture was allowed to react at 0 °C for 8 h to form a polyamic acid gel (m-DABPAA). After the gel was fully de-bubbled, it was scraped onto a clean glass plate. The thickness of the liquid film was controlled by adjusting the effective height of the scraper to control the thickness of the PI film. The liquid film was imidised in a high-temperature vacuum-drying oven with the following temperature ramp: room temperature to 100 °C (1 h), 100 to 200 °C (1 h), 200 to 300 °C (1 h), 300 to 350 °C (0.5 h), and natural cooling from 350 °C to room temperature. Then, the PI film was peeled from the glass plate. IR (KBr, *v*, cm^−1^): 1775 and 1717 (C=O stretching), 1364 (C–N stretching), 1100~700 (Ar–H stretching). Anal. calcd for C_23_H_11_N_3_O_5_: C, 67.48; H, 2.71 and N, 10.27; found: C, 67.39; H, 2.75 and N, 10.33. DABPI was prepared from commercial DABA and PMDA by a similar process as M-DABPI.

### 2.6. Molecular Simulation

Biovia Materials Studio software was employed to perform simulations in this study using the COMPASS (Condensed-phase Optimized Molecular Potentials for Atomistic Simulation Studies) forcefield [21,22]. Initially, polymer chains containing 25 repeat units were built and geometry optimization was performed. Then, a periodic model of the PI, comprising of 5 polymer chains in cubic unit cell, was constructed and the total energy of the system was minimized using smart minimizing method. Annealing was performed by the NPT (constant number of particles (N), pressure (P) and temperature (T)) dynamics procedure through heating and cooling the system at 1 atm in the temperature range of 300 to 1000 K in steps of 50 K. Interactions of non-bond, van der Waals and electrostatic forces, were calculated using an atom-based summation method and an Ewald summation method, respectively. The annealed cell was later put through a stage-wise equilibration procedure. First the cell was heated to 1000 K, and then the temperature was decreased in several stages to 600 K in steps of 100 K. After this, the temperature was decreased in several stages to 400 K in steps of 50 K and then decreased to 300 K in steps of 25 K. Each stage consisted of two consecutive NVT (constant number of particles (N), volume (V) and temperature (T)) and NPT runs at 1 atm and a specific temperature. The aim of the procedure was to obtain a refined system that would relax at the experimental density of the amorphous polymer at 1 atm and 300 K. Finally, the cell was relaxed by consecutive NVT (at 300 K) and NPT dynamics (at 1 atm and 298 K) to ensure that a constant density has been reached. Two criteria were used to determine the equilibrium of the system: (1) The density of the system remained stable for a long time; (2) the fluctuation of energy was lower than 10% [23]. The plots of density and energy versus simulation time in the last NPT for PI systems are shown in Figures S1 and S2, respectively. It can be seen that the PI systems have reached equilibrium states. The equilibrated densities of Kapton and FAPPI systems are 1.47 and 1.50 g/cm^3^, respectively, which are fairly close to the experimental densities (1.51 and 1.53 g/cm^3^). In all runs, the Nosé method was used for temperature control [24]. In NPT runs, the pressure was controlled by Berendsen’s method. During these simulations, the cutoff for the nonbonded interactions was taken as 15.5 Å.

The fractional free volume (FFV), hydrogen bonds, radius of gyration, radial distribution functions (RDFs), local mobility of polymer molecular chains, gas diffusion coefficient, and solubility coefficient of the two PI materials were investigated via molecular simulation and are elaborated in the Supporting Information.

## 3. Results and Discussion

### 3.1. Synthesis and Characterisation of Monomers and Polyimides

**Scheme 1:**
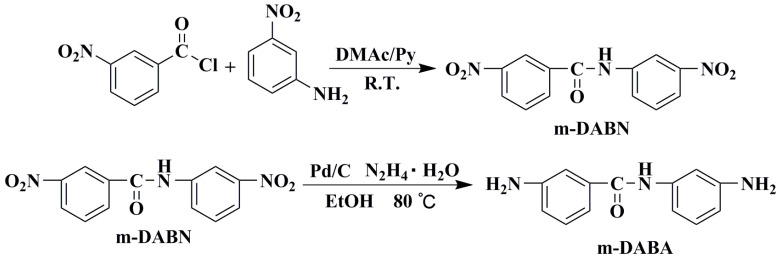
m-DABA synthesis route.

**Figure 2:**
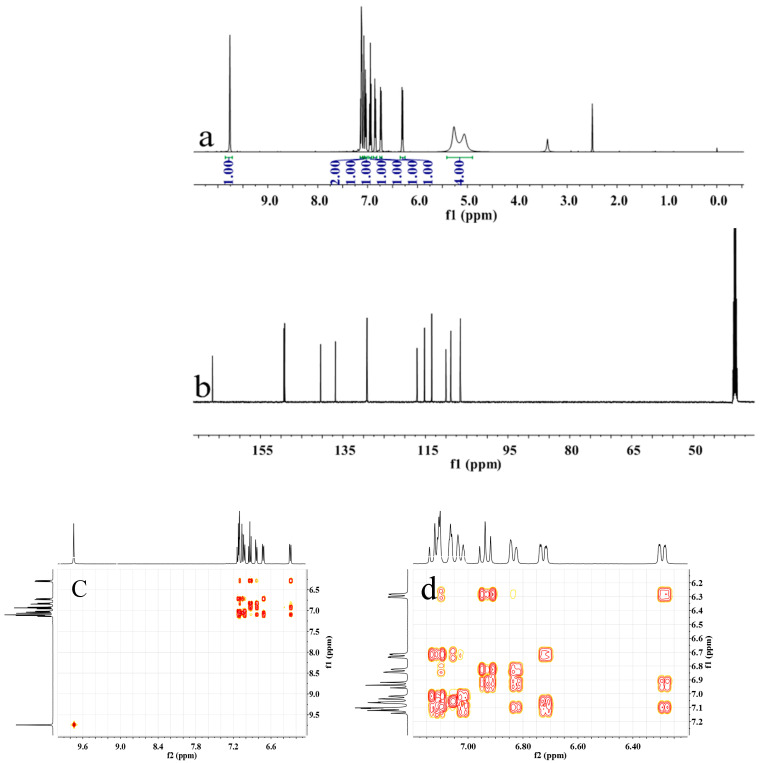

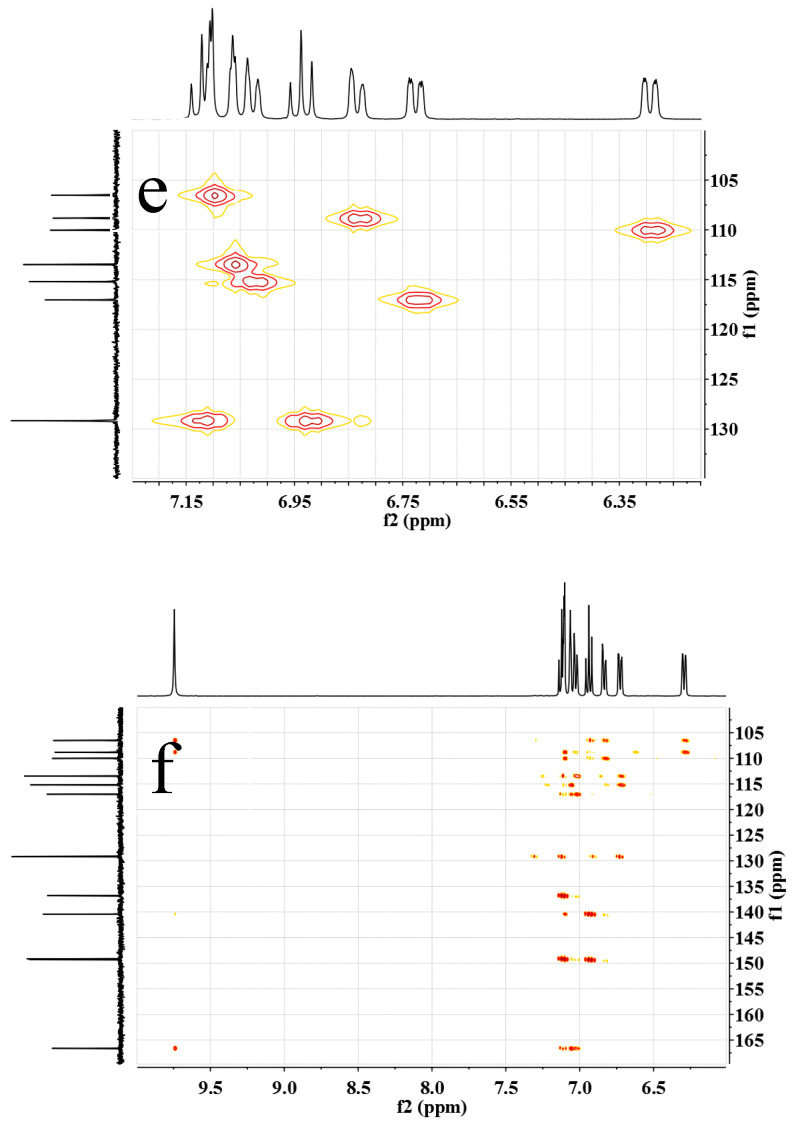
(**a**) ^1^H NMR, (**b**) ^13^C NMR, (**c**,**d**) H–H COSY, (**e**) C–H QC and (**f**) C–H BC spectra of m-DABPI in DMSO-*d*_6_.

Dinitro-intermediate m-DABN was obtained by reacting m-nitrobenzoyl chloride and m-nitroaniline as raw materials at room temperature for 1 d. m-DABN was catalytically reduced using Pd/C and hydrazine hydrate to yield the dianiline monomer m-DABA with high purity. The synthesis route is shown in Scheme 1. m-DABA and the intermediate were characterised by NMR, FT-IR, elemental analysis, and mass spectrometry, and the results verified the success of the monomer synthesis. Figures S3 and S4 in the Supplementary Materials present the m-DABN NMR and MS spectra, respectively. Figure S5 presents the IR spectra of the two structures and presents the NMR spectrum of m-DABA. The NMR spectra of m-DABA are shown in Figure 2 and elemental analysis results are described in the experimental section.

**Scheme 2:**
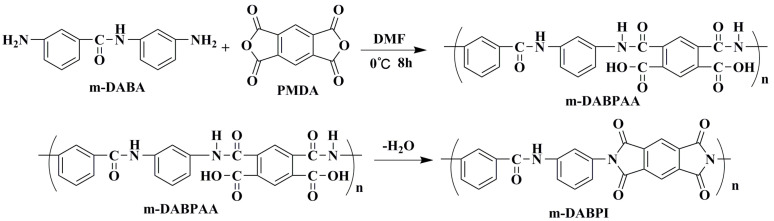
Polyimide synthesis route.

The high-viscosity precursor m-DABPAA was prepared by polymerising the same amount of m-DABA and PMDA at low temperatures. These materials were then thermally dehydrated and cyclised to yield PI (m-DABPI). The synthesis route is shown in Scheme 2. As m-DABPI did not dissolve in organic solvents, gel permeation chromatography (GPC) was used to determine the average molecular weight and molecular weight distribution of m-DABPAA. *M_w_* was 2.15 × 10^5^, and the polydispersity index (*M_w_/M_n_*) was 1.92, indicating that the synthesized PI had a high molecular weight with a narrow distribution. Figure S5 presents the FT-IR spectrum of m-DABPI, which exhibits characteristic absorption peaks of imide groups at 1.364 cm^−1^ (stretching of C–N) as well as at 1775 and 1.13 cm^−1^ (stretching of carbonyl groups), in contrast to the IR spectrum of m-DABA. This indicates that the reaction between m-DABA and PMDA was successful and imidisation was complete.

### 3.2. Thermal and Mechanical Properties

The thermal properties of m-DABPI were investigated using DMA, TMA, and TGA; the resultant curves are shown in Figure S6, and the results are summarised in Table 1. The 5% and 10% weight-loss temperatures (*T_d5%_* and *T_d10%_*) of m-DABPI reached 498 °C and 522 °C respectively (Figure S6a), and the glass transition temperature (Tg) reached 364 °C (Figure S6b). The thermal expansion coefficient of m-DABPI was 21.7 ppm·K^−1^ at 50–300 °C, (Figure S6c). Moreover, the PI had a tensile strength and tensile modulus of 131 MPa and 4.1 GPa, respectively, showing excellent mechanical properties. The image of bent m-DABPI film (Figure S7) indicates that the PI film had good flexibility. The introduction of amide moieties led to a strong interchain force in the m-DABPI film, thereby giving it good thermal properties and excellent mechanical performance.

### 3.3. Barrier Properties

Barrier properties were compared between the present m-DABPI and the previously reported para-substituted PI (DABPI) as summarised in Table 2. The introduction of an amide moiety in the monomer led to excellent barrier properties in both PI materials but especially in m-DABPI, which had a water vapour transmission rate (WVTR) of 2.8 g·m^−2^·d^−1^ and an oxygen transmission rate (OTR) of 3.3 cm^3^·m^–2^·d^–1^, amounting to 54.9% and 41.8% of the DABPI counterparts, respectively. This may be because the meta-substituted monomer better facilitated compact packing of the PI molecular chains, thereby reducing gas permeability and improving barrier properties. At the same time, compared with the traditional Kapton film, changing the molecular structure like m-DABPI to improve the barrier performance of PI will greatly reduce the inorganic and organic layers required by flexible display, and reduce the complexity and cost of production [14,15,16].

### 3.4. Aggregation Structures Analysis

**Figure 3:**
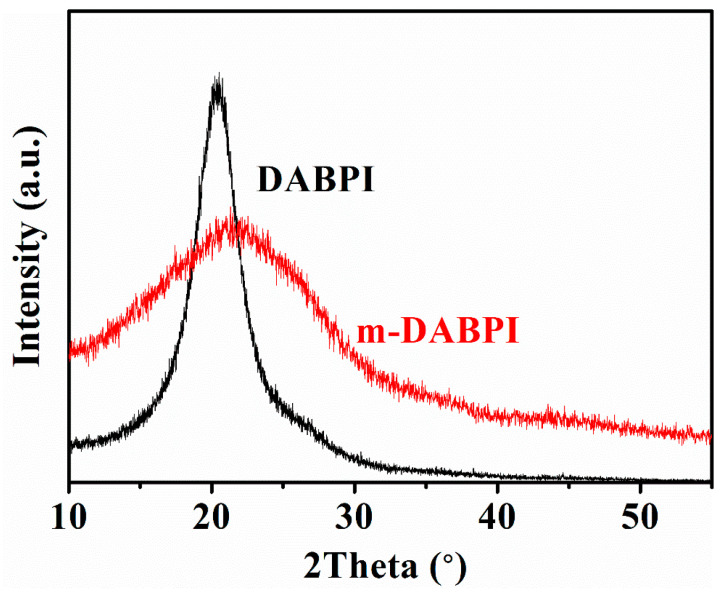
WAXD curve of DABPI and m-DABPI films.

The two PI materials were characterised via wide-angle X-ray diffraction (WAXD) to reveal their molecular chain-packing characteristics, thereby illustrating the impact of stereoisomerism on PI barrier properties. The WAXD curves of the two PI materials are shown in Figure 3. The molecular chain spacing in the two PI materials was calculated using the peak diffraction angle according to Bragg’s equation [25], with the results shown in Table 3. The peak diffraction angle of DABPI was 20.5°, and the corresponding chain spacing was 4.33 Å. For m-DABPI, the peak diffraction angle was 21.7°, and the chain spacing was only 4.09 Å. Further, the density of the two PI materials was tested, and the results are shown in Table 3. m-DABPI had the larger density at 1.53 g·cm^−3^. The smaller chain spacing and higher density of m-DABPI indicate that the molecular chains of meta-substituted PI were packed more tightly in this monomer.

### 3.5. Hydrogen Bond Analysis

**Figure 4:**
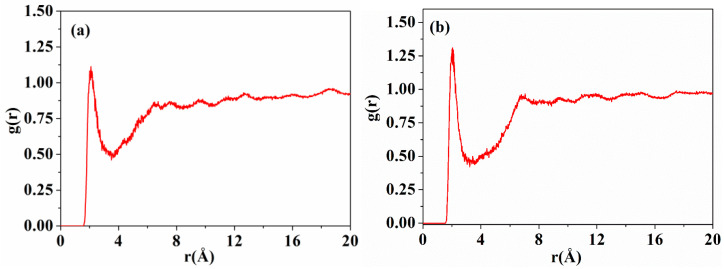
The RDFs of the hydrogen atoms of ‒HN‒ and oxygen atoms of O=C‒ for DABPI (**a**) and m-DABPI (**b**). RDFs:Radial distribution function usually refers to the distribution probability of other particles in space given the coordinates of a given particle (how far away from the given particle).

**Figure 5:**
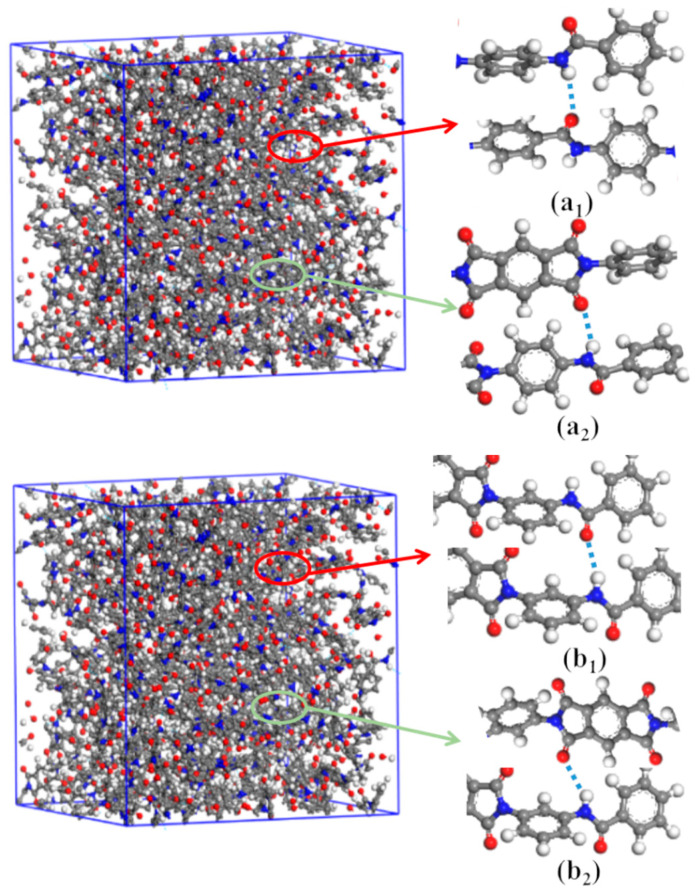
H-bonds in the simulation cell of DABPI (**a_1_**,**a_2_**) and m-DABPI (**b_1_**,**b_2_**). H-bonds between ‒HN‒ and O=C‒ in amide groups (**a_1_**,**b_1_**) and between ‒HN‒ and O=C‒ in imide rings (**a_2_**,**b_2_**).

Inter-chain forces are crucial to molecular chain packing in polymers. The radial distribution functions (RDFs) of DABPI and m-DABPI were calculated through molecular simulation to investigate the hydrogen-bonding force in the two PI materials. Theoretically, two types of hydrogen bonds may exist in each PI material, namely hydrogen bonds formed by the ‒NH‒ in amide moieties with the O=C‒ in amide moieties and with the O=C‒ in imide rings. It has been reported that hydrogen-bonding forces exist only over the range of 0 to 3.1 Å, while van der Waals forces can exist at distances greater than 3.1 Å [26]. The RDF diagrams of the two PI materials (Figure 4) exhibited obvious peaks near 1.9 Å and 2.1 Å, indicating that the two PI materials were likely to form hydrogen bonds (shown in Figure 5). m-DABPI had a higher number of hydrogen bonds, 60, in the simulation box than did DABPI (Table 3). Thus, the results of these simulation-based calculations showed that m-DABPI had greater cohesive energy density than did DABPI (Table 3). More hydrogen bonds and greater cohesive energy density lead to stronger inter-chain forces, which in turn promote closer packing of molecular chains.

### 3.6. Free Volume and Cavity Size Distribution Analysis

**Figure 6:**
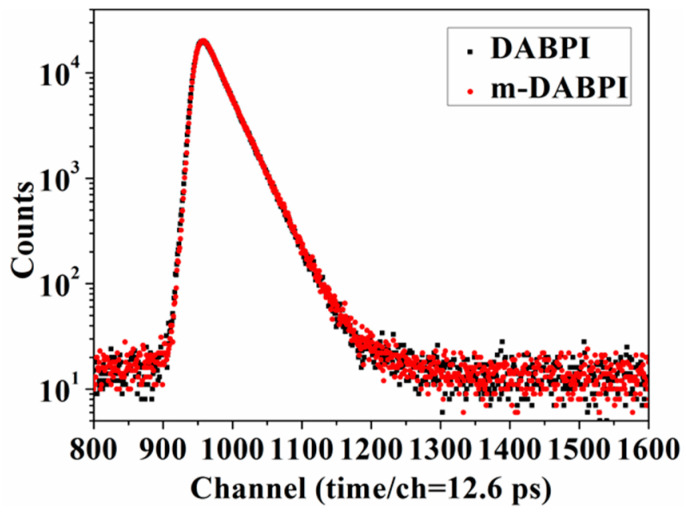
Positron lifetime spectra of DABPI and m-DABPI films.

The free volume of DABPI and m-DABPI was characterised by PALS. Table 4 lists the positron lifetime components of the PI materials while Figure 6 presents the corresponding positron lifetime curves. These results were considered alongside previous research and relevant reports to calculate the free volume radius (R), free volume size (V_f2_), and fractional free volume (FFV) [17,27]. (Table 4). m-DABPI had smaller V_f2_ and FFV values than did DABPI. The average V_f2_ was 41.63 Å^3^ in DABPI and 38.79 Å^3^ in the m-DABPI film, corresponding to FFVs of 7.28% and 6.77%, respectively. The smaller V_f2_ and FFV were attributed to the closer packing of molecular chains in the meta-substituted PI.

**Figure 7:**
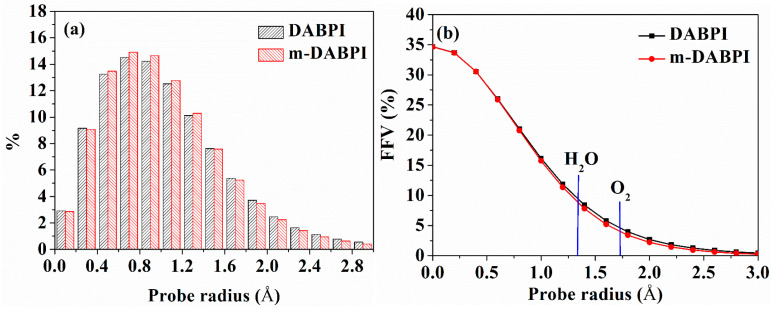
Distributions of void radii (**a**) and fractional free volume (**b**) as a function of probe radius in DABPI and m-DABPI. The kinetic radii of O_2_ and H_2_O are indicated by vertical lines.

Molecular simulation could elucidate the free volume connectivity and pore size distribution of polymers, thereby promoting a deeper understanding of PI barrier properties. Figure 7a shows the relationship between the void radius (0 to 3 Å) and void percentage obtained through molecular simulation. Given that permeating molecules can only diffuse through voids with radii larger than their dynamic radius (which is 1.325 Å and 1.73 Å for H_2_O and O_2_, respectively), voids with radii greater than 1.2 Å are considered effective. The peak radius in Figure 7a is about 0.8 Å, which is the radius of most voids. Such a small radius implies that the regions available for small molecule diffusion in the two PI materials were small. The percentage of voids with radii greater than 1.2 Å was lower in m-DABPI than in DABPI, indicating that the region for small molecule diffusion in m-DABPI was smaller than that in DABPI. These observations confirm that molecular chain linking via the meta-position served to improve PI barrier properties compared with the linking achieved via the para-position.

**Figure 8:**
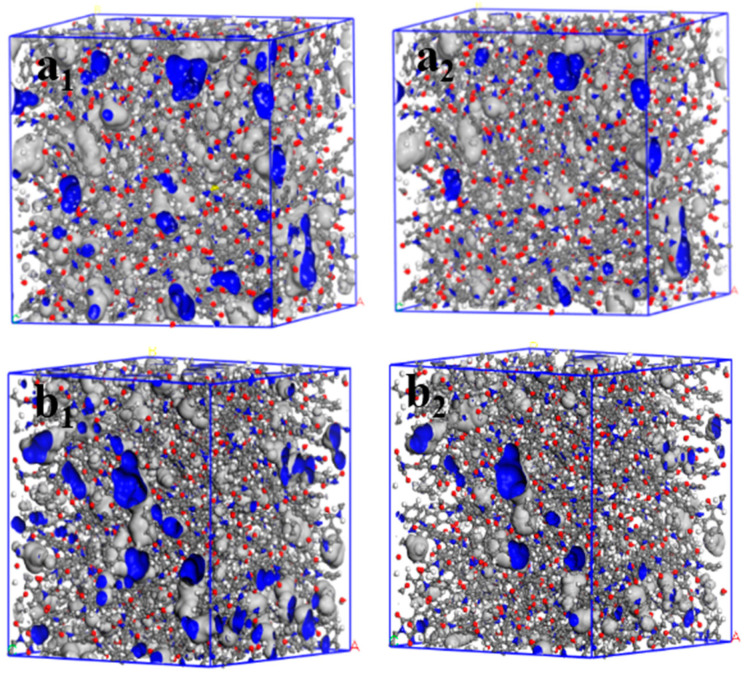
Three-dimensional representation of H_2_O and O_2_ accessible volume in (**a1**,**a2**) DABPI, and (**b1**,**b2**) m-DABPI (grey: Van der Waals surface; blue: Connolly surface; a1 and b1: probe radius of 1.325 Å; a2 and b2: probe radius of 1.73 Å).

In addition, the relationship between the radius of a spherical probe and FFV values was investigated (Figure 7b). The FFV of the two PI materials gradually decreased as the spherical probe radius increased, being lower in m-DABPI than in DABPI. When the spherical probe radius was equal to the dynamic radius of H_2_O and O_2_, the corresponding FFV was denoted as *FFV* (O_2_) and *FFV* (H_2_O), respectively. Table 4 presents the FFVs of the two PI materials when H_2_O and O_2_ molecules were separately used as the probe. The results obtained via both molecular probes showed that compared with DABPI, m-DABPI had a smaller free volume, which indicated that m-DABPI was less conducive for the permeation of small molecules. Figure 8 presents the simulated morphology of free volume sensed via separate H_2_O and O_2_ probes (the blue areas denote voids). As shown in Figure 8, m-DABPI has fewer blue areas and a smaller free volume than DABPI, which is consistent with the more compact packing of molecular chains in m-DABPI.

### 3.7. Local Mobility of Polymer Chains

**Figure 9:**
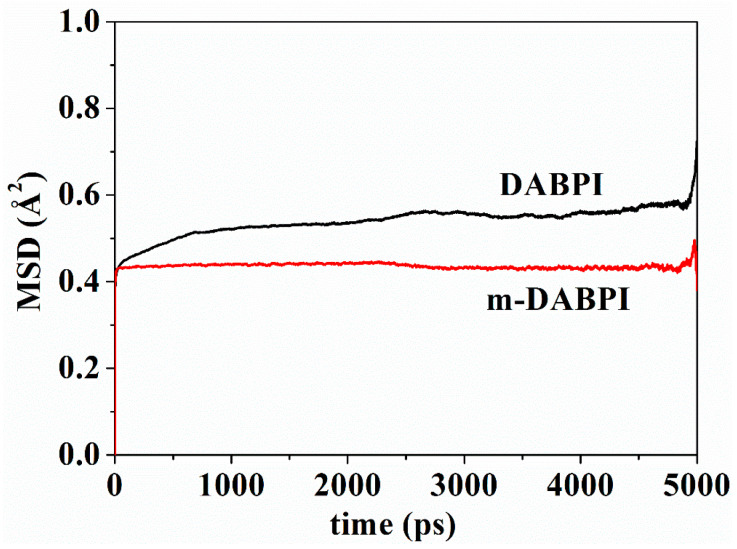
MSD of polymer chains as a function of time in DABPI and m-DABPI.

Small molecules diffuse in a polymer film by jumping from one void to another through the channel transiently formed via molecular chain motion [28]. Therefore, the local mobility of molecular chains is crucial for gas barrier properties. The higher the local mobility of molecular chains, the easier the gas diffusion [29,30]. Mean square displacement (MSD) in the two PI materials was separately calculated via molecular simulation. Figure 9 presents MSD as a function of time up to 5000 ps in the two PI materials. A larger MSD value indicates greater molecular chain mobility. Compared with DABPI, it is more difficult for m-DABPI to form a diffusion channel for small molecules due to its weaker chain mobility. This property contributes to m-DABPI’s stronger barrier properties.

### 3.8. Gas Transport Behaviour

Molecular simulation of gas diffusion and dissolution in the two PI materials was performed to gain deeper insights into their gas permeability.

#### 3.8.1. Gas Diffusion

**Figure 10:**
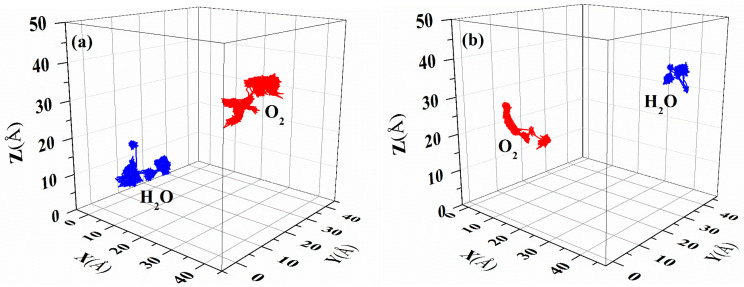
Representative trajectories of O_2_ and H_2_O in (**a**) DABPI and (**b**) m-DABPI.

The diffusion of gas molecules in a polymer film can be approximated as the random movement of small molecules in the film [31]. Figure 10 illustrates the diffusion trajectories of H_2_O and O_2_ as representative small molecules over a period of 10 ns in the two PI materials, and Figure S8 presents the corresponding $\left|r_{i}\left(t\right)-r_{i}\left(0\right)\right|$ values through which the displacement curve of m-DABPI is translated. Figure 10 and Figure S8 clearly indicate that the two types of gas molecules have shorter trajectories and displacements in m-DABPI than in DABPI, which is attributable to the fact that the molecular chains of m-DABPI are more compactly packed and have a smaller free volume, thereby limiting small molecule diffusion.

**Figure 11:**
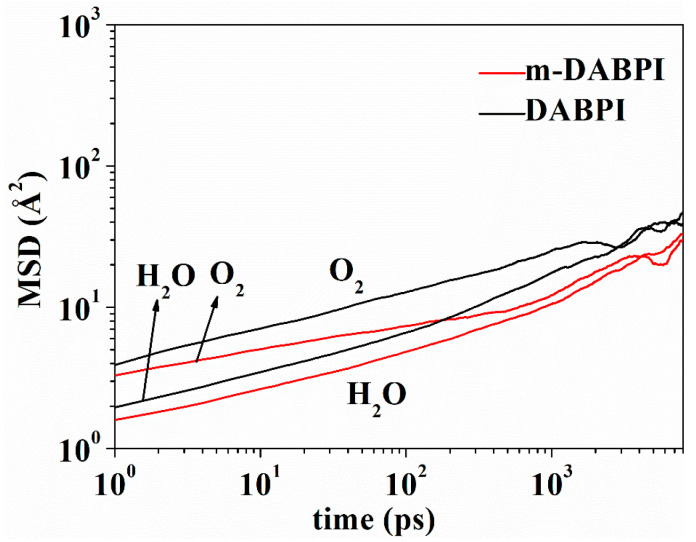
MSD of O_2_ and H_2_O in DABPI and m-DABPI as a function of time.

The relationship between MSD and time was plotted on logarithmic scales (Figure 11). The diffusion coefficient of the penetrating molecules in the PI materials was derived according to the corresponding Einstein equation [32]. The results (Table 5) showed that the diffusion coefficient was lower in m-DABPI than in DABPI. This was attributable to the smaller free volume and thereby more limited gas diffusion in m-DABPI, which was consistent with the diffusion trajectories. However, H_2_O, which has a smaller dynamic radius than does O_2_, had a smaller diffusion coefficient in this study, which was attributed to the fact that the two PI materials contained strong hydrogen-bonding forces and thereby limited the diffusion of H_2_O [33]. As shown above, the substitution position impacts the gas diffusion properties of PI, with meta-substitution being unfavourable for small molecule diffusion.

#### 3.8.2. Gas Solubility

**Figure 12:**
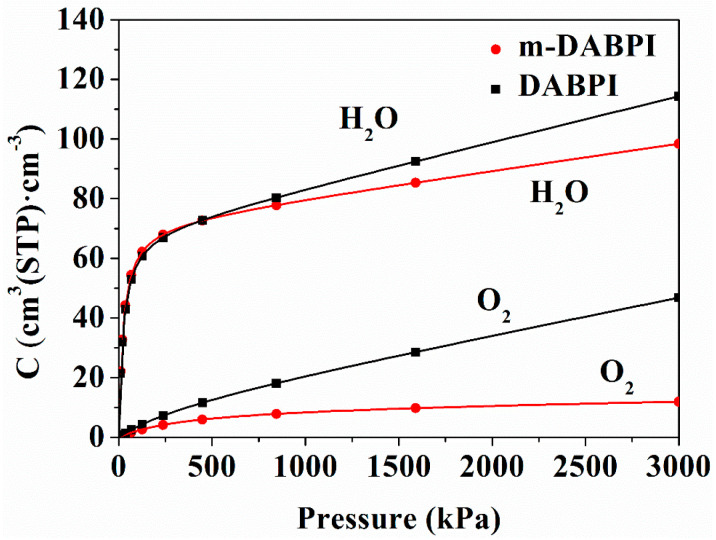
Adsorption isotherms of O_2_ and H_2_O in DABPI and m-DABPI.

Molecular simulation of the adsorption isotherms of H_2_O and O_2_ in the two PI films was performed to gain deeper insights into their dissolution behaviour in the materials. the solubility describes the concentration of the gas inside a polymer at equilibrium with the gas at a partial pressure and is often described by the dual-mode sorption theory; As shown by the four simulated adsorption isotherms in Figure 12, rapid adsorption occurs under low pressure, which mainly occurs in inter-chain voids, while slow desorption occurs under high pressure, which mainly occurs in the intra-chain free volume. These two processes coincide with Langmuir-mode adsorption and Henry-mode adsorption, respectively [34]. Moreover, solubility coefficients were derived from the adsorption isotherms, and the results (Table 5) showed that m-DABPI had smaller solubility coefficients than DABPI. This is because m-DABPI has a smaller free volume and therefore fewer adsorption sites.

#### 3.8.3. Gas Permeability

Permeability coefficients (P) were calculated as the product of diffusion coefficients (D) and solubility coefficients (S) according to the dissolution-diffusion mechanism [35]. The results (Table 5) showed that compared with DABPI, m-DABPI had smaller permeability coefficients, which was attributed to its smaller diffusion coefficients and solubility coefficients. The results showed a trend consistent with that of the experimentally derived permeability coefficients in this study, indicating the high reliability of the simulated results. In summary, therefore, PI synthesised from meta-substituted monomers has better barrier properties, because meta-substitution is more favourable for the close packing of molecular chains and therefore leads to a smaller free volume and reduced mobility.

## 4. Conclusions

The present study designed and synthesised the meta-substituted dianiline monomer m-DABA as a stereoisomer of the previously investigated para-substituted dianiline monomer DABA, and polymerised m-DABA with PMDA to prepare PI in a process similar to that employed in the previous study. The impact of substitution position on the barrier properties of polyimide was investigated. The results showed that the introduction of amide moieties led to good barrier properties in the two polyimide materials, especially the meta-substituted polyimide. Moreover, an in-depth analysis was performed via WXAD, PALS, and molecular simulation, revealing that meta-substitution better promoted the close packing of molecular chains in the polymer to yield a small free volume, thereby inhibiting to some extent the dissolution and diffusion of permeating molecules in the polymer film. Further, the molecular chains of m-DABPI had poor mobility, which was not conducive to the diffusion of permeating molecules. Taken together, these factors resulted in lower permeability coefficients for m-DABPI, which are indicative of better barrier properties. In addition, m-DABPI maintained good thermal and mechanical properties. These findings have significant implications for the structural design importance of high-barrier materials which could advance the development of flexible display technology.

## Figures and Tables

**Table 1:** Thermal and mechanical properties of the m-DABPI film.

Polyimide	*T_g_*^a^ (°C)	*T_d5%_* (°C)	*T_d10%_* (°C)	CTE ^b^ (ppm·K^−1^)	Tensile Strength (MPa)	Tensile Modulus (GPa)
m-DABPI	364	498	522	21.7	131 ± 3.0	4.1 ± 0.2

^a^ Measured by DMA; ^b^ CTE within the range of 50–300 °C.

**Table 2:** Barrier properties of DABPI and m-DABPI films.

PIs	WVP (g·mil·m^−2^·d^−^^1^)	OP (cm^3^·mil·m^−2^·d^−^^1^)	WVTR (g·m^−2^·d^−^^1^)	OTR (cm^3^·m^−2^·d^−^^1^)
Kapton ^a^	/	/	209.8 ± 3.1	657.3 ± 3.8
DABPI ^a^	3.6 ± 0.2	5.6 ± 0.2	5.1 ± 0.3	7.9 ± 0.3
m-DABPI	2.0 ± 0.2	2.3 ± 0.2	2.8 ± 0.2	3.3 ± 0.2

^a^ The barrier properties values were obtained from Liu [18].

**Table 3:** Physical properties of DABPI and m-DABPI.

PIs	Density (g·cm^−3^)	2θ (°)	*d*-Spacing (Å)	CED ^b^ (J/cm^3^)	N ^c^ (H-Bonds)
DABPI ^a^	1.51	20.5	4.33	557	58
m-DABPI	1.53	21.7	4.09	612	60

^a^ The density, 2θ, *d*-spacing values were obtained from Liu [18]. ^b^ 1 mol of condensate per unit volume is the energy needed to overcome forced intermolecular vaporization. ^c^ Number of hydrogen bonds in the simulation cells.

**Table 4:** Analyzed data of the positron lifetime and simulated *FFV* values in the DABPI and m-DABPI films.

PIs	*τ_1_* (ns)	*I_1_* (%)	*τ_2_* (ns)	*I_2_* (%)	*R* (Å)	*V_f2_* (Å^3^)	*FFV* ^a^	*FFV*^b^ (O_2_)	*FFV*^b^ (H_2_O)
DABPI	0.18	2.5	0.370	97.2	2.15	41.63	7.28	4.52	9.60
m-DABPI	0.19	2.6	0.368	97.0	2.10	38.79	6.77	3.95	9.03

^a^*FFV* was determined by PALS. ^b^ Free volume parameters calculated by simulations; *FFV* (O_2_) and *FFV* (H_2_O) values are based on probe radii of 1.73 Å and 1.325 Å, respectively.

**Table 5:** Simulated diffusion coefficients (D), solubility coefficients (S), and permeability (P) for O_2_ and H_2_O in DABPI and m-DABPI.

PIs	D ^a^		S ^b^		P ^c^	
	H_2_O	O_2_	H_2_O	O_2_	H_2_O	O_2_
DABPI	1.7	4.82	0.41	0.045	0.70	0.22
m-DABPI	1.2	3.19	0.39	0.038	0.47	0.12

^a^ Units: (10^−8^ cm^2^/s). ^b^ Units: (cm^3^(STP) cm^−3^·cmHg^−1^). ^c^ Units: (10^−8^ cm^2^·cm^3^(STP). s^−1^·cm^−3^·cmHg^−1^).

## Data Availability

The data used to support the findings of this study are available from the corresponding author upon request.

## Supplementary Materials

The following are available online at https://www.mdpi.com/article/10.3390/ma14092097/s1: Figure S1: Plots of density versus simulation time in the NPT simulation for DABPI and m-DABPI. Figure S2: Plots of energy versus simulation time in the NPT simulation for (a) DABPI and (b) m-DABPI. Figure S3: ^1^H NMR (a), ^13^C NMR (b) and MS (c) spectra of m-DABN. Figure S4: MS spectrum of m-DABA. Figure S5: FT-IR spectra of m-DABN, m-DABA and m-DABPI. Figure S6: TGA (a), DMA (b) and TMA (c) curve of m-DABPI film. Figure S7: Photo image of the flexible m-DABPI film. Figure S8: Displacement of O_2_ and H_2_O from their initial positions in DABPI and m-DABPI.
