# Supplementary material for: Impact of Backbone Amide Substitution at the Meta- and Para-Positions on the Gas Barrier Properties of Polyimide
Source: Materials (Basel). 2021 Apr 21;14(9):2097. doi: 10.3390/ma14092097 (PMC8122393; doi:10.3390/ma14092097)
Supplement: Supplementary file 1 [file materials-14-02097-s001.zip › materials-1169026-supplementary.pdf]

materials

Supplementary Materials

Impact of Backbone Amide Substitution at the Meta- and Para-Positions on the Gas Barrier Properties of Polyimide

Qian Wen [1], Ao Tang [2], Chengliang Chen [2], Yiwu Liu [2,*], Chunguang Xiao [1], Jinghua Tan [2] and Duxin Li [1,*]

1 State Key Laboratory of Powder Metallurgy, Central South University, Changsha 410083, China; wenqian0088@126.com (Q.W.); chunguang4715@126.com (C.X.); liduxin6404@csu.edu.cn (D.L.)
2 National and Local Joint Engineering Center of Advanced Packaging Materials R & D Technology, Key Laboratory of Advanced Packaging Materials and Technology of Hunan Province, School of Packaging and Materials Engineering, Hunan University of Technology, Zhuzhou 412007, China; tangao1234@163.com (A.T.); chenchengliangc@163.com (C.C.); tjh@hut.edu.cn (J.T.)
* Correspondence: liuyiwu@hut.edu.cn (Y.L.); liduxin6404@csu.edu.cn (D.L.)

Citation: Wen, Q.; Tang, A.; Chen, C.; Liu, Y.; Xiao, C.; Tan, J.; Li, D. Impact of Backbone Amide Substitution at the Meta- and Para-Positions on the Gas Barrier Properties of Polyimide. *Materials* **2021**, *13*, 2097. https://doi.org/10.3390/ma14092097

Academic Editor: Volker Abetz

Received: 18 March 2021
Accepted: 7 April 2021
Published: 21 April 2021

Publisher's Note: MDPI stays neutral with regard to jurisdictional claims in published maps and institutional affiliations.

Copyright: © 2021 by the authors. Submitted for possible open access publication under the terms and conditions of the Creative Commons Attribution (CC BY) license (http://creativecommons.org/licenses/by/4.0/).

1. Instrumentation

1.1. Instrumentation

All Nuclear Magnetic Resonance spectra (NMR) were recorded on a Bruker ARX400 MHz spectrometer (Bruker Corporation, Fallanden, Switzerland). Samples were prepared as solution of 5~15 mg of compound in 0.5 mL of deuterated dimethyl sulfoxide (DMSO) using tetramethylsilane (TMS) as the internal reference. Mass spectra were measured on a AcquityUPLC/UPC2/Xevo G2-XS QTOFMS (Waters Corporation, Milford, MA, USA). Elemental analysis was carried out on a Vario EL cube Elemental Analyzer (Elementar Corporation, Langenselbold, Germany). Infrared spectra were recorded on a Nicolet iS10 Fourier-transform infrared (FT-IR) spectrometer (Thermo Fisher Scientifific, Waltham, MA, USA). The molecular mass of the poly(amic acid) (PAA) was estimated by gel permeation chromatography on multi-angle laser light scattering (GPC-MALLS) system (Wyatt Technology Corporation, Santa Barbara, CA, USA). DMF is the eluent with flow rate of 1 mL/min and test temperature of 50 °C. WAXD were recorded by a Rigaku, Ultema III X-ray diffractometer (Rigaku Corporation, Tokyo, Japan) using a Cu Kα radiation. Density was obtained with an ALFA MIRAGE SD-200L electronic density balance(Alfa Mirage Corporation, Osaka, Japan).Thermogravimetric analyses (TGA) were performed with a TA thermal analyzer (TGA55, TA instruments, New Castle, DE, USA) under N_2 with a heating rate of 20 °C/min from 50 to 800 °C, and heated under flowing nitrogen (40 mL/min). The dynamic mechanical (DMA) spectra of the samples were obtained by using TA thermal analyzer (DMA Q850, TA instruments, New Castle, DE, USA). The specimens were analyzed in tensile mode at a constant frequency of 1 Hz, amplitude of 20 μm, and a temperature range from 25 to 375 °C at a heating rate of 5 °C/min. Thermal mechanical analysis (TMA) was used to study the coefficient of thermal expansion of the film with a heating rate of 5 °C/min from 25 °C to 350 °C by TMA Q400 instrument (TA instruments, New Castle, DE, USA)

under nitrogen. Tensile test was performed on samples cut from 35~50 μm thick sheet and tested using SANS CMT6103 instrument (New Sansi Material Testing Co., Ltd. Shenzhen, China) according to GB/T16421-1996. The specimen size is 10×100 mm, Jaw separation is 50 mm. Jaw speed was first set to 2 mm/min, when elongation reached 1 mm, the Jaw speed was changed to 20 mm/min.The oxygen permeability of a PI film was measured by Mocon (Minneapolis, MN, USA), in accordance with ASTM-D 3985, using an OX-TRAN 2/21 ML instrument at 23 °C and 0% RH. The moisture vapor permeability was measured using the model PERMATRAN-W® 3/33 of the Mocon Corporation (Indio, CA,USA) at 90% RH and 37.8 °C according to ASTM F-1249. The sample of 75 μm was fixed on an aluminum foil with the testing area of 5 cm^2.Positron lifetime measurements were performed as follows: two identical samples with dimension of 1.5 × 10 × 10 mm^3 were sandwiched with a 10 μCi ^{22}Na positron source. The ^{22}Na nucleus emits a 1.28 MeV γ-ray simultaneously (within a few ps) with the positron. The positron lifetime is determined from the time delay between the emission of the birth gamma (1.28 MeV) and one of the 0.511 MeV annihilation photons. Lifetime measurements were carried out using a fast-fast coincidence system with a time resolution of about 210 ps and a channel width of 12.6 ps. We have analyzed the lifetime spectra with the help of the data processing programs PATFIT. Before analyzing each spectrum, the positron source components (377 ps/11.75%, 1.04 ns/0.17%) were subtracted. The variance of the fits was around

2. Details of Molecular Simulation

2.1. Plots of Density, Energy Versus Simulation Time in the NPT Simulation for DABPI and m-DABPI.

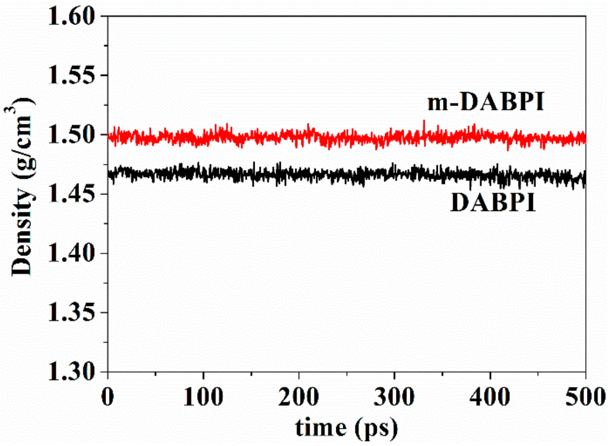

Figure S1. Plots of density versus simulation time in the NPT simulation for DABPI and m-DABPI.

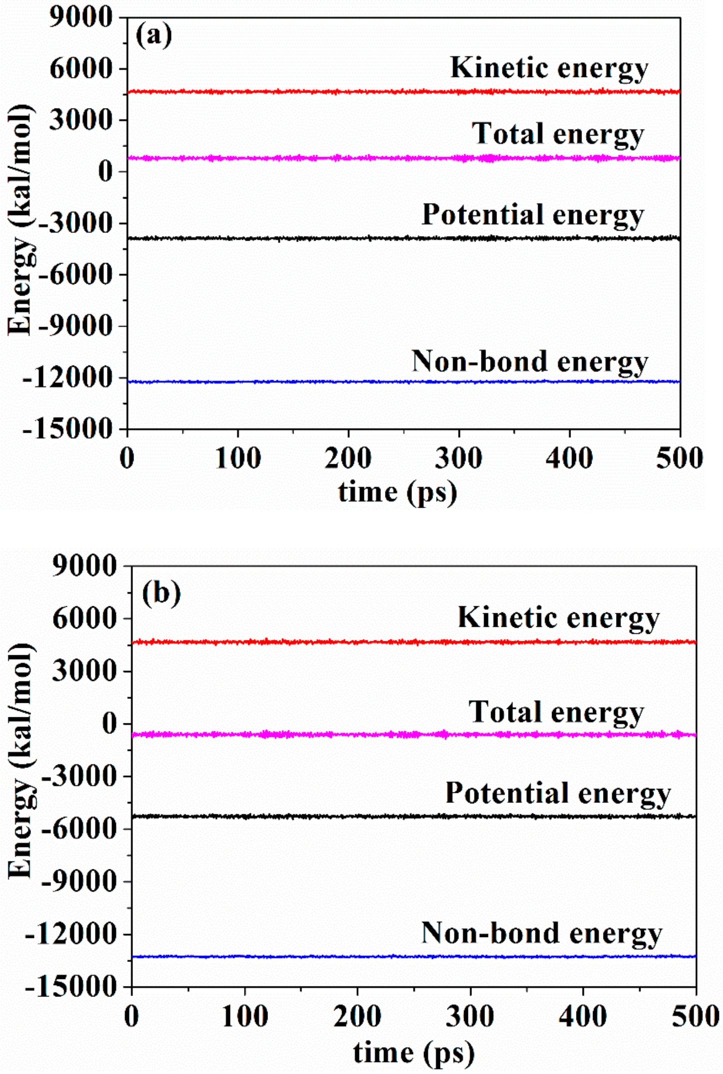

Figure S2. Plots of energy versus simulation time in the NPT simulation for (**a**) DABPI and (**b**) m-DABPI.

2.2. Free Volume

The free volume was determined by a grid scanning method using the Connolly task. The void distribution was estimated by a method previously used for micro-crystalline materials [1,2]. Specifically, the simulation cell was divided into three-dimensional fine grids with a size of approximately 0.25 Å. The void size at a grid was determined as the diameter of the maximum cavity that encloses the grid and additionally has no overlap with any polymer atom.

2.3. Radial Distribution Functions

The radial distribution functions (RDF) refer to a measure of the probability that, given the presence of an atom at the origin of an arbitrary reference frame, there will be an atom with its center located in a spherical shell of infinitesimal thickness at a distance r from the reference atom. The RDF was calculated by the average of the static relationship of every given pair of particles AB using the following equation [3]:

$$g_{AB}(r) = \frac{\langle n_{AB}(r)\rangle}{4\pi r^2 \Delta\rho_{AB}} \quad (1)$$

where $\langle n_{AB}(r)\rangle$ is the average number of atom pairs between r and $r + \Delta r$, and $\Delta\rho_{AB}$ is the density of atom pairs of type AB.

2.4. Diffusion Coefficients

The diffusivity of gas molecules through PI was estimated by first inserting ten molecules of each gas into the equilibrated simulation box. Then, minimization of the potential energy was performed using ''smart minimizing method'' run. After this, the cell was put through annealing and stage-wise equilibration procedures using the same parameters as described before. The resulting structure was then equilibrated by NVT and NPT simulations at 298 K in order to ensure that its minimized total energy remained approximately constant with respect to the simulation time. An NVE simulation of the system was performed for 10,000 ps at room temperature. The diffusion coefficients can be calculated by means of the Einstein relation [4,5]

$$\mathrm{D} = \frac{1}{6N} \lim_{t\to\infty} \frac{d}{dt} \langle \sum_{i}^{N} |r_i(t) - r_i(0)^2|\rangle \quad (2)$$

where N is the number of penetrants, $r_i(0)$ and $r_i(t)$ are the initial and final positions of the center of mass of penetrant i over the time interval t, and $\langle |r_i(t) - r_i(0)|^2\rangle$ is the averaged mean-square displacement (MSD) of the penetrant. The diffusion coefficient was determined from the slope of MSD versus time data. In this work, MSD of H_2O and O_2 were calculated from the trajectories of ten penetrant molecules in the PI microstructures.

2.5. Determination of Local Mobility of Polymer Chains

The backbone MSD of PIs was determined to investigate the local mobility of PI chains. An NVE simulation of the equilibrated system was performed for 5000 ps.

2.6. Sorption Isotherm

The equilibrated cell was used for grand canonical Monte Carlo (GCMC) simulations employing the standard Metropolis algorithm using the "Sorption Isotherm" module [6]. Both the polymer framework and the penetrant molecules were treated as rigid bodies. The degrees of freedom of the system were accordingly specified by the center-of-mass position and orientation of the molecules. Metropolis sampling was used for inserting or deleting permeant molecules as well as accepting or rejecting their translational and rotational configurational moves. The COMPASS force field and force field assigned partial charges on atoms were used. A VTμ simulation was performed at each fixed pressure and 298 K. The pressure of the penetrant gas was varied from 10 to 3000 kPa. For each pressure value, 10^5 equilibration steps were first performed to ensure proper relaxation of the polymer chains in response to the insertion of the penetrant molecule, following which 10^6 steps of production run were carried out. The sorption isotherm can be obtained in the form of a plot of the

concentration of sorbed gas, C, as a function of pressure at constant temperature. The solubility coefficient, S, is then obtained from the limiting slope of the sorption isotherm at zero pressure as [7]

$$S = \lim_{p \to \infty} (C/p) \quad (3)$$

where C is in units of cm^3(STP)/cm^3(polymer) and p is pressure.

3. Characterization and Properties of Monomers and Polyimides

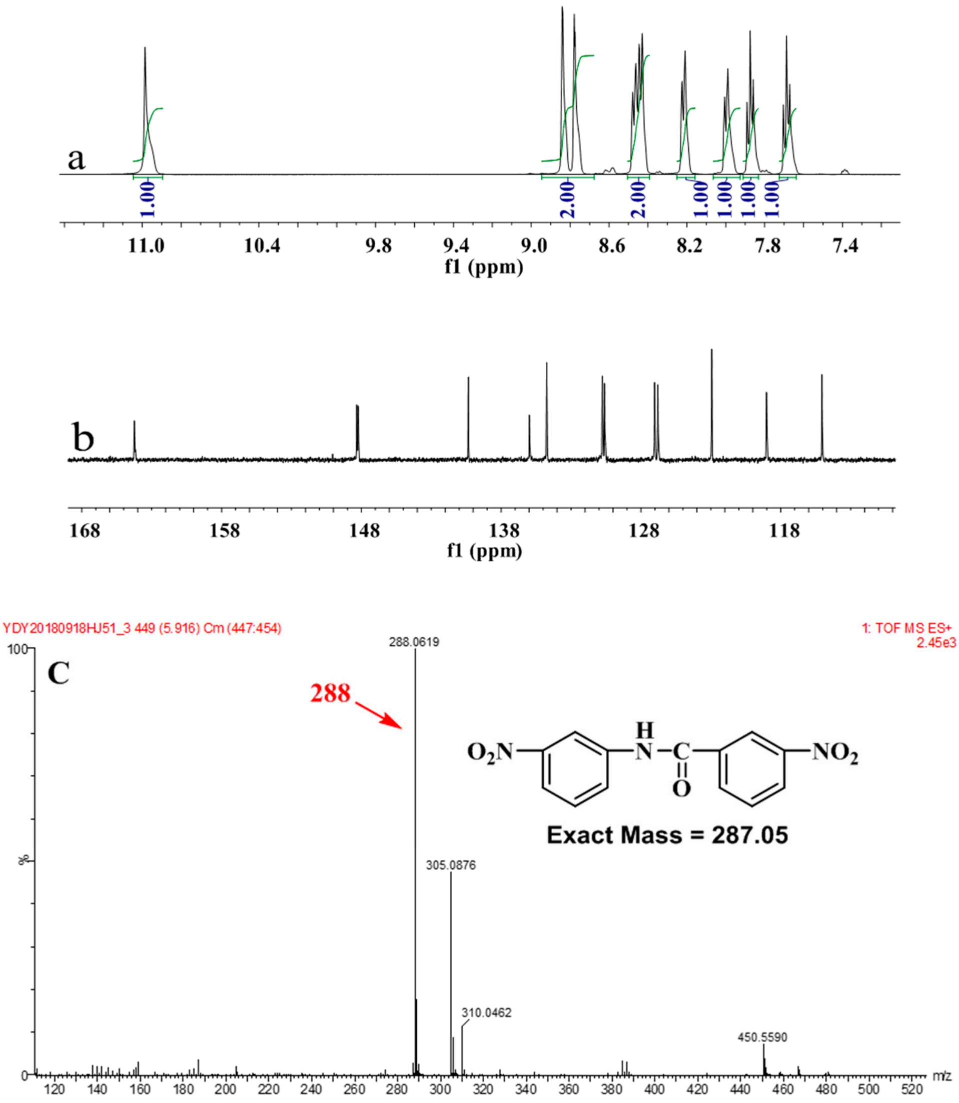

Figure S3. ^{1}H NMR (**a**), ^{13}C NMR (**b**) and MS (**c**) spectra of m-DABN.

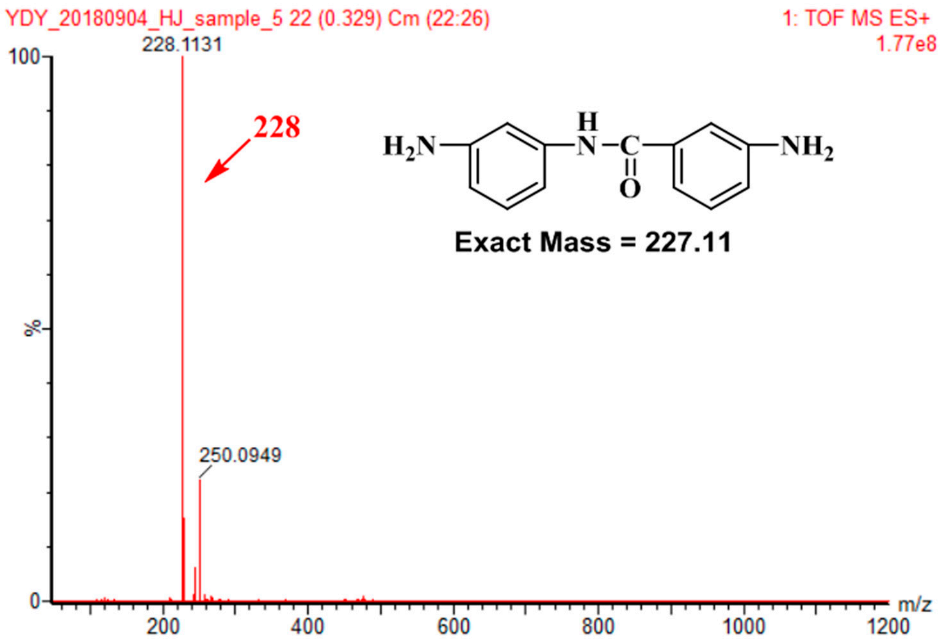

Figure S4. MS spectrum of m-DABA.

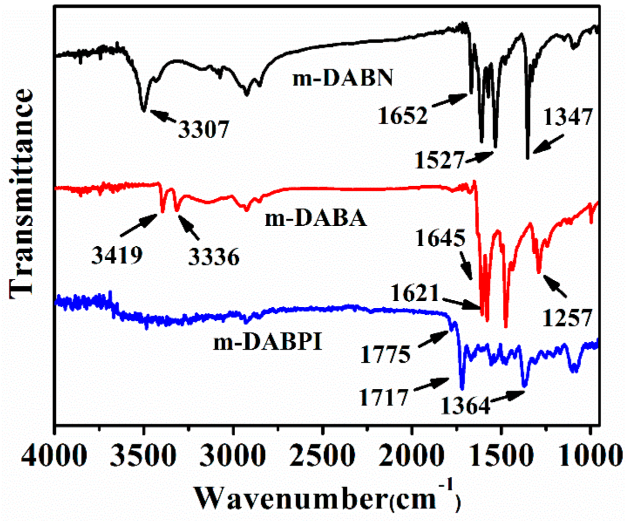

Figure S5. FT-IR spectra of m-DABN, m-DABA and m-DABPI.

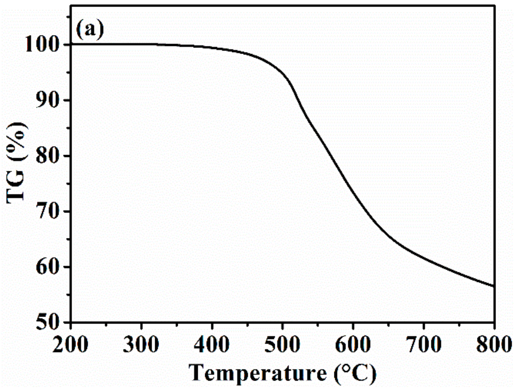

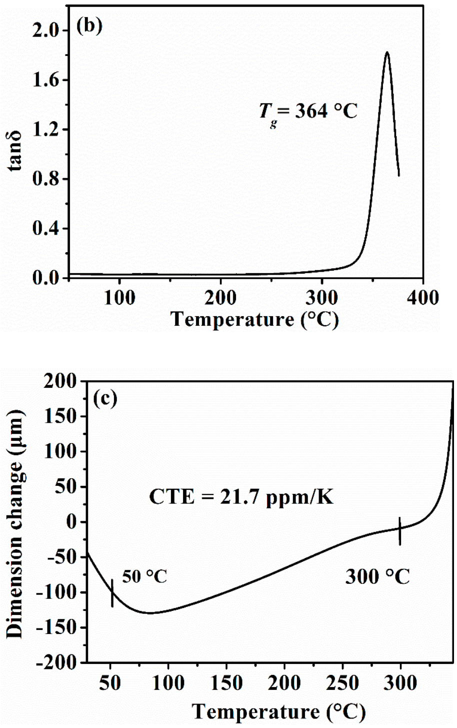

Figure S6. TGA (**a**), DMA (**b**) and TMA (**c**) curve of m-DABPI film.

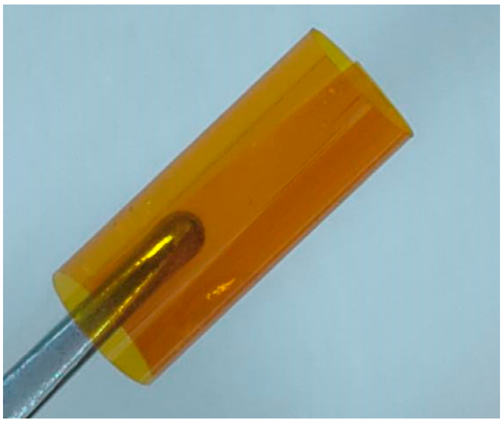

Figure S7. Photo image of the flexible m-DABPI film.

4. Gas Diffusion

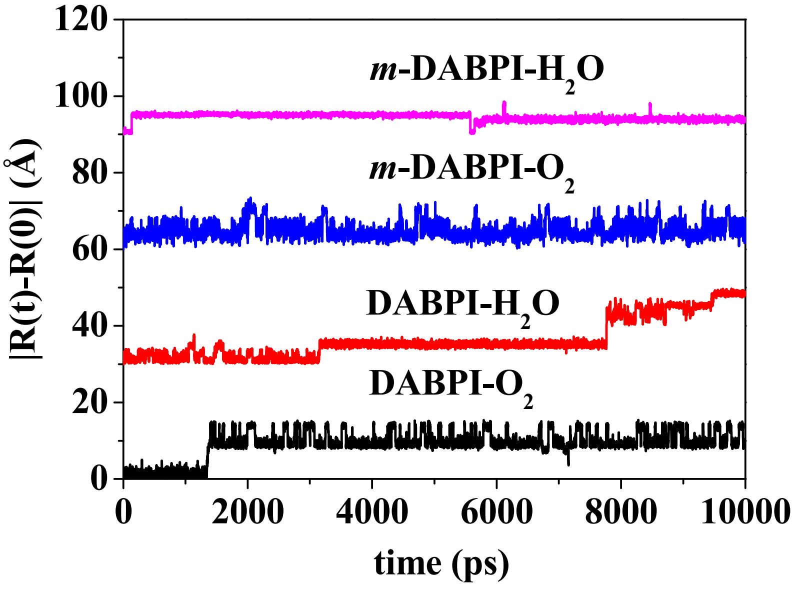

Figure S8. Displacement of O_2 and H_2O from their initial positions in DABPI and m-DABPI.

References

1. Ban, S.; Vlugt, T.J. Zeolite microporosity studied by molecular simulation. *Mol. Simul.* **2009**, *35*, 1105–1115, doi:10.1080/08927020802660614.
2. S. Bhattacharya and K. E. Gubbins, Fast method for computing pore size distributions of model materials. *Langmuir* **2006**, *22*, 7726–7731.
3. Zeng, J.; Zhang, Y.; Dai, Y.; Chen, S. Molecular dynamics simulation of nitrobenzene in heterocyclic ionic liquids. *J. Mol. Liq.* **2014**, *198*, 274–279, doi:10.1016/j.molliq.2014.07.022.
4. Pant, P.V.K.; Boyd, R.H. Molecular-dynamics simulation of diffusion of small penetrants in polymers. Macromolecules **1993**, *26*, 679–686, doi:10.1021/ma00056a019.
5. Cuthbert, T.R.; Wagner, N.J.; Paulaitis, M.E.; Murgia, G.; D'Aguanno, B. Molecular Dynamics Simulation of Penetrant Diffusion in Amorphous Polypropylene: Diffusion Mechanisms and Simulation Size Effects. *Macromolecules* **1999**, *32*, 5017–5028, doi:10.1021/ma980997e.
6. Akkermans, R.L.; Spenley, N.A.; Robertson, S.H. Monte Carlo methods in Materials Studio. *Mol. Simul.* **2013**, *39*, 1153–1164, doi:10.1080/08927022.2013.843775.
7. Fried, J.; Sadat-Akhavi, M.; Mark, J. Molecular simulation of gas permeability: poly(2,6-dimethyl-1,4-phenylene oxide). *J. Membr. Sci.* **1998**, *149*, 115–126, doi:10.1016/s0376-7388(98)00151-3.
